# From arterial hypertension complications to von Hippel-Lindau syndrome diagnosis

**DOI:** 10.1186/s13052-015-0158-y

**Published:** 2015-08-13

**Authors:** Sylwia Kozaczuk, Iwona Ben-Skowronek

**Affiliations:** Department of Paediatric Endocrinology and Diabetology, Medical University in Lublin, Poland, ul. Prof. A. Gebali 6, 20-093 Lublin, Poland

**Keywords:** Arterial hypertension, Pheochromocytoma, von Hippel-Lindau syndrome

## Abstract

Von Hippel-Lindau syndrome is a rare, genetically based, autosomal dominant disorder. Its course is accompanied by the development of multiple neoplasms with the following tumours diagnosed most commonly in the central nervous system haemangioblastoma, clear cell renal cell carcinoma, phaeochromocytomas, pancreatic islet tumours, and endolymphatic sac tumours. Additionally, renal and pancreatic cystadenomas and epididymal cystadenomas have been diagnosed in males and cystadenomas of the broad ligament of the uterus have been diagnosed in females.

The following paper presents the diagnostic way in a boy with vision disorders as the first symptom. Hypertension retinopathy and extremely elevated blood pressure were observed during ophthalmologic consultation. Complications of arterial hypertension were confirmed by echocardiography, which diagnosed hypertension cardiomyopathy. Hypertension retinopathy was confirmed by optical coherence tomography. Examinations performed in the neurology, cardiology, and finally endocrinology indicated a bilateral phaeochromocytoma as the cause of arterial hypertension. Moreover, some genetic investigations showed a mutation in the VHL ex.1 p.Y112 C gene responsible for the hereditary form of phaeochromocytoma which confirmed von Hippel-Lindau syndrome. After surgical treatment of phaeochromocytoma the patient needed careful management according to the surveillance protocol for von Hippel-Lindau disease.

## Background

Von Hippel-Lindau syndrome (retinal-cerebellar angiomatosis) is a rare, genetically based, autosomal dominant disorder. Its course is accompanied by with the development of multiple neoplasms with the following tumours diagnosed most commonly in the central nervous system haemangioblastoma, clear cell renal cell carcinoma, phaeochromocytomas, pancreatic islet tumours and endolymphatic sac tumours. Additionally renal and pancreatic cystadenomas and epididymal cystadenomas have been diagnosed in males and cystadenomas of the broad ligament of the uterus in females [1–3].

The incidence of the syndrome is estimated at ca. 1/36 000 of live births [4]. The disease is associated with a mutation in both alleles of the vhl gene located on the short arm of chromosome 3. Until now over 150 mutations responsible for the development of the syndrome have been identified [5–11].

VHL disease is a hereditary cancer syndrome characterized by the incidence of multiple vascular tumours. The syndrome is caused by inactivation of the von Hippel–Lindau protein (pVHL) [6]. Loss of the functional VHL protein results in a high level of HIF, which causes increased production of VEGF, PDGF, and TGF- alpha. This explains cell growth and proliferation of microvascular vessels. HIF also contributes to overproduction of thyrosine hydroxylase and catecholamines in phaeochromocytoma. It is the cause of inhibition of apoptosis of neural crest cells and development of phaeochromocytoma and paraganglioma [1, 6, 7]. The syndrome has been divided into 2 types depending on the risk of phaeochromocytoma development.

The spectrum of von Hippel-Lindau syndrome includes Chuvash polycythaemia, otherwise referred to as familial erythrocythemia type 2. This is a rare form of the disease not associated with tumours and its incidence is particularly high in the Chuvash population inhabiting the Volga River region [1, 6].

Tumours associated with the VHL syndrome are typically bilateral and multifocal but rarely malignant [2, 7, 12–16]. Unlike sporadic neoplasms, they affect younger patients [14].

 The first symptoms of the disease are most often related to haemangioblastmas in the retina, cerebellum, and other regions of the central nervous system [17]. Patients suffer from weakness, pain in the arms and legs, back ache, headaches, numbness, and dizziness. Polycythaemia caused by overproduction of erythropoietin is usually diagnosed.

MRI is used for diagnosis of haemangioblastomas. Patients presenting with VHL syndrome for more than 10 years should be examined with MRI once a year. Small and asymptomatic tumours only need to be carefully observed. Symptomatic tumours located in the cerebellum or in proximity of optic nerves as well as large haemangioblastomas should be removed neurosurgically or treated with the gamma knife. Haemangioblastomas and postoperative morbidity are the major causes of physical disability in VHL patients.

Retinoblastoma develops in the early foetal period and at the age of <10 year. Majority of patients do not present any symptoms. Retinal haemangioblastomas are diagnosed by means of ophthalmoscopy or fluorescent angiography. The treatment of choice involves laser photocoagulation in the early stage of the disease. Removal of the affected eye should be considered in tumours associated with loss of vision.

Another cause of vision defects is retinopathy connected with arterial hypertension in the development of phaeochromocytoma [18–20]. In the course of VHL syndrome, phaeochromocytomas develop most frequently in children and young patients. They can be located in both the adrenal glands and paraganglia [14, 15]. The most common symptoms of phaeochromocytoma comprise drenching sweats, headaches, bouts of anxiety and agitation and palpitations, as well as skin pallor, which is frequently regarded as a pathognomonic symptom [7, 16–25]. According to some authors, paroxysmal or chronic arterial hypertension is a basic symptom of phaeochromocytomas. Literature of the subject provides information that arterial hypertension in children is usually chronic, unlike in adults, who suffer from periodic episodes of hypertension [24, 25]. Very high chronic levels of arterial tension may result in occurrence of complications, e.g. hypertensive cardiomyopathy or retinopathy with retinal detachment [26]. The diagnosis of sporadic and inherent phaeochromocytomas is based on biochemical and imaging analyses. Hormonally active tumours can be detected by measurement of levels of catecholamines and methoxycatecholamines in serum or in 24-hour urine collection (the method of choice) [21]. High sensitivity of this method is associated with the fact that catecholamines (adrenaline and noradrenaline) are secreted by the tumour periodically and their elevated levels can only be detected during episodes when a substantial amount of the substances are secreted. In contrast, the level of methoxycatecholamines is permanently elevated due to the internal conversion of catecholamines to methoxycatecholamines within the tumour [27–41]. Phaeochromocytomas in the course of VHL syndrome are assumed to secrete primarily noradrenaline and methoxynoradrenaline. In turn, sporadic phaeochromocytomas and tumours developing in the course of MEN 2 or NF1 syndromes secrete adrenaline, noradrenaline, and their metabolites [36, 42–44]. Laboratory tests often reveal carbohydrate metabolism disorders, leucocytosis, and hypocalcaemia. The latter condition can be caused by increased sequestration of calcium ions within the tumour which utilises them while releasing catecholamines [44]. Laparoscopic partial adrenalectomy, which usually involves glucorticosteroid substitution, is the method of choice in phaeochromocytoma treatment in the paediatric population [32].

Apart from nodular lesions, numerous cystic lesions located in different regions can occur in VHL syndrome. Two cases of epididymal cystadenomas as the first signs of VHL syndrome have been reported in the literature of the subject [34], but pancreatic and renal cystadenomas may also occur [15, 30]. These types of lesions, compound cystadenomas in particular, require intensive supervision, since there is probability of solid mass development with a clear cell renal cell carcinoma component [15, 31].

In the case of diagnosis of VHL syndrome components, genetic tests should be performed in the patient and family members. Qualification can be based on criteria specified by Massachusetts General Hospital [33].

**Fig. 1:**
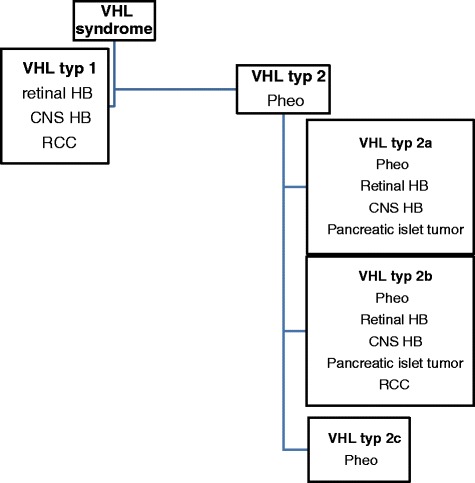
Classification of VHL syndrome

Genetic tests should be carried out in:

1. any individuals with two VHL-associated lesions: HB, RCC, Pheo, endolymphatic sac tumour, epididymal or adnexal papillary cystadenoma, pancreatic cystadenomas, and neuroendocrine tumours.
2. any individuals with one or more of the following: CNS HB, Pheo or paraganglioma, endolymphatic sac tumour, and epididymal papillary cystadenoma.
3. any individuals with > RCC diagnosed <20 years, bilateral or multiple RCC, in multiple pancreatic serous cystadenoma and neuroendocrine tumour, pancreatic cyst, and any VHL associated lesion. Classification of VHL syndrome  is showed in Fig. 1.

## Case presentation

**Fig. 2:**
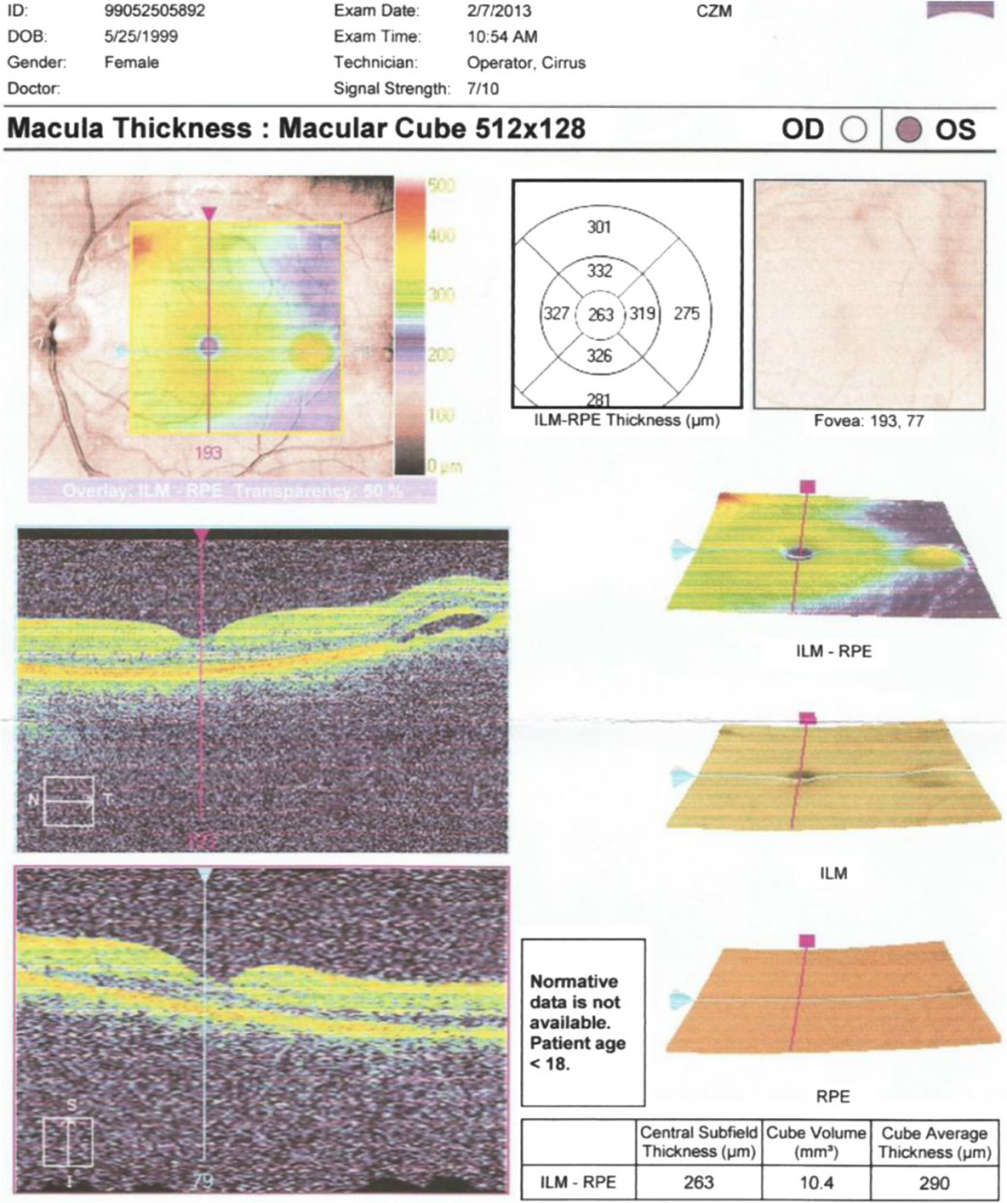
Hypertensive retinopathy in optical coherence tomography in a 14-year-old boy. The odema of the macula and haemorrgage to the retina is showed

The patient - a boy aged 14 with uncomplicated perinatal history (pregnancy 1, delivery 1, 38/39 hbd, 3400 g b.w., length 54 cm, Apgar score 9) underwent ophthalmic examination due to bilateral scotoma. The eye examination showed increased intraocular pressure and regions with retinal detachment, which in combination with elevated values of arterial hypertension (180/98 mmHg) indicated hypertensive retinopathy (Fig. 2). The patient was referred to the Department of Neurology for further diagnosis due to periodic headaches and dizziness. The boy was in a relatively good condition on admission and no signs of neurological syndromes were revealed by physical examination; however, increased arterial hypertension of approx. 180/135 mmHg was diagnosed. The contrast computed tomography scan of the head showed a fluid-dense, non-contrast enhanced 3.5 × 2.5 × 4.0 cm area in the anterior part of the temporal lobe with thinned lamellae of adjacent bones; the image corresponded to an arachnoid cyst. One focus was located subcortically within the right temporal lobe (1 cm) and two foci were shown in the right and left cerebellar hemispheres (up to 1, 4 cm). According to the neurosurgical consultation the phenomenon needed observation. No pathological signs were exhibited by the bilateral optical nerves in the intraconic and chiasm sections. The 3, 8 × 2 cm arachnoid cyst previously shown by CT located near the anterior pole of the right frontal lobe was also confirmed.

**Fig. 3:**
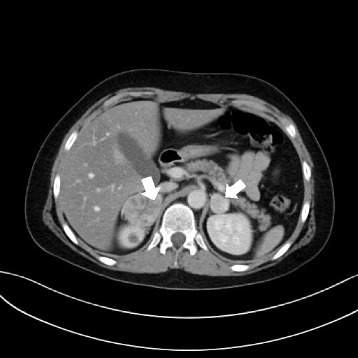
Bilateral nodular lesions in the right and left adrenal glands (phaeochromocytoma) in abdominal computed tomography

Since the arterial hypertension persisted at very high values (194/147 mmHg, 189/153 mmHg) and tachycardia ca.120/min. occured, the patient was referred to the Department of Paediatric Cardiology in fair-serious condition. Echocardiographic examination showed signs of hypertensive cardiomyopathy in the form of grade II mitral regurgitation and left ventricular hypertrophy without obstruction of the outflow tract. The patient’s condition deteriorated sharply during hospitalization, and the boy presented with severe headaches with vomiting, tongue numbness, and paresthesia of the upper and lower left limbs. Since CNS bleeding in the course of the hypertensive crisis was suspected, an immediate head CT scan was performed, which showed signs of cerebral oedema. Antihypertensive drugs and decongestants, i.e. captopril, amlodipine, carvedilol, furosemide, and 20 % Mannitol 100 ml i.v., were administered. Abdominal ultrasound examination showed a ca. a 41 × 35 mm hypoechoic tissue lesion in the right adrenal gland without visible vascularization in colour and power Doppler which demonstrated no visible pathological lesions in the left adrenal gland. The Doppler ultrasound of the aorta and renal arteries did not show significant abnormalities. To confirm typical phaeochromocytoma symptoms, the patient’s history was taken again; it provided information that the patient had suffered from periodic bouts of anxiety accompanied by skin pallor and excessive sweating. Ultrasound examination results combined with the clinical manifestations suggested a phaeochromocytoma in the right adrenal gland; therefore, the patient was referred to the Department of Paediatric Endocrinology and Diabetology for further diagnosis and treatment. Laboratory tests revealed an elevated glucose level (143 mg/dl), hypokalemia (3,19 mmol/l), and hypocalcaemia (8,2 mg/dl). The 4-fold assays of catecholamine levels in blood serum showed a very high noradrenaline content accompanied by a normal level of adrenaline; in 24-hour urine collection, the catecholamine and methoxycatecholamine levels were substantially elevated (Table 1). In addition, the levels of neuron-specific enolase and calcitonin were measured and thyroid ultrasound was performed to exclude neuroblastoma and MEN 2. The results of these examinations were normal. The imaging diagnostics comprised an additional abdominal CT scan, which confirmed the presence of a ca. rl 50 × ap 40 × cc 52 mm nodular lesion in the right adrenal gland and a similar but smaller ca. 24-mm-diameter lesion in the left adrenal gland (Fig. 3).

**Table 1:** Catecholamines and their metabolites in patients with VHL syndrome before and after surgical treatment of phaeochromocytoma

	Before surgical treatment		After surgical treatment	Norm ranges	Units
Metanephrins in 24-hour urine collection	2.68	2,68	0,30	<1	mg/24 h
Adrenalin in 24-hour urine collection	45,6	81,7	9,0	1,3-14,5	μg/24 h
Noradrenalin in 24-hour urine collection	4047,0	3105,3	18,8	8,3-51,1	μg/24 h
Dopamin in 24-hour urine collection		643,8	448,6	75,2-433,8	μg/24 h
Adrenalin in serum	76	58	x	30,0-90,0	ng/l
Noradrenalin in serum	>5000,0	>5000,0	x	165,0-460,0	ng/l

The lesions were substantially pronounced in the arterial phase. Additionally, a fluid-dense cystic area with a diameter of ca. 15 mm was detected in the upper part of the right kidney., Antihypertensive treatment was maintained in the Department of Endocrinology; the patient’s clinical condition improved and his arterial tension was satisfactorily stabilized. After premedication with the Plummer solution, the patient was examined by ^131 I^-MIBG isotopic scintigraphy of adrenergic receptors. Examination of the entire body was performed after 48 and 72 h, and abdominal SPECT imaging was used 72 h after intravenous radioisotope injection. Regions exhibiting increased accumulation of the tracer were visible in both adrenal glands, but the area was larger in the right adrenal gland. After full diagnostics and premedication, the patient received lateral transperitoneal bilateral adrenalectomy in the Department of Surgery.

In the right adrenal gland, histopathological examination showed a 5-cm diameter solid-cystic tumour, grey-cherry red in section, limited to the gland.

Additionally, another solid tumour with a diameter of 1, 5 cm was localized. Otherwise, the adrenal gland was unchanged. Microscopic image: phaeochromocytoma; immunohistochemistry: chromogranin A (+), synapthophysin (+); proliferative index Ki67 3–4 %; acc.to the PASS system: 1 pt (capsule invasion). The left adrenal gland: a 2.7-cm diameter encapsulated tumour, grey-cherry red in section, limited to the gland. Microscopic image: phaeochromocytoma; Immunohistochemistry: proliferative index Ki67: <2 %; acc.to the PASS system: 0 pt.

The patient has been receiving hydrocortisone replacement therapy.

 Due to a suspicion of the hereditary form of phaeochromocytoma in the patient, a genetic test was carried out. It showed a mutation in the VHL ex.1 p.Y112 C gene responsible for the hereditary form of phaeochromocytoma and confirming von Hippel-Lindau syndrome. A further genetic test was prescribed to detect hereditary syndromes with the phaeochromocytoma component in other family members.

Currently, 20 months after the surgery, the patient feels good and does not report any alarming symptoms. Measurements of arterial tension and the heart rate carried out at home show normal results. Follow-up abdominal and thyroid ultrasonography is normal; isotopic analysis performed 8 months after the surgery (^131- I^ MIBG scintigraphy) did not reveal any regions of abnormal accumulation of the tracer. The level of catecholamines and methoxycatecholamines in the control 24-h urine collection was normal (Table 1). Ophthalmic examinations showed significant regression of the changes described earlier. The patient’s vision has improved substantially and there are no signs of scotoma. Follow-up MRI of the CNS did not reveal abnormalities besides the presence of the arachnoid cyst. The lesions detected previously may have resulted from the developing encephalopathy. Slight muscular hyperthrophy of the left ventricle persists.

## Conclusion

Recent data from the National Health and Nutrition Survey indicates that 10 % of children and adolescents suffer from prehypertension and 4 % have hypertension [41,45–46]. Pheochromocytomas occur in about 0.05 % to 0.1 % of patients with sustained hypertension. Pheochromocytoma is a rare, secondary cause of juvenile hypertension which usually occurs since the age of 10 years. Recommendations for the early diagnosis of hypertension in childhood involve screening for hypertension in all children over the age of 3 at every visit and ordering laboratory evaluation, echocardiography, and renovascular imaging for all children given a diagnosis of hypertension [44].

Up to 20 % of pheochromocytomas are diagnosed in children. Most of them are functional tumors, and clinical presentation includes symptoms related to catecholamine hypersecretion andr tumor mass effect. Increasingly, pheochromocytomas are identified during presymptomatic screening in children with genetic syndromes: multiple endocrine neoplasia type 2, von Hippel-Lindau disease, and the paraganglioma syndromes [36].

The case presented in this case study shows the complexity of VHL syndrome which causes diagnostic difficulties. The first visible sign of the disease is arterial hypertension, which should be diagnosed by a physician during prophylactic investigations, otherwise it may lead to complications. The diagnosis of tumours typical of VHL syndrome, especially in a paediatric patient, necessitates more comprehensive diagnostics and application of genetic tests in the patient and family members as well as intensive supervision of carriers of the mutated gene. This approach facilitates early diagnosis of tumour lesions and frequently successful treatment thereof.

## Consent

Written informed consent was obtained from the patient for publication of this case report and any accompanying images. A copy of the written consent is available for review by the Editor-in-Chief of this journal.

## Abbreviations

VHL: von Hippel-Lindau
Pheo: Pheochromocytoma
Retinal HB: Retinal hemangioblastoma
CNS Hb: Central nervous system hemangioblastoma
RCC: Clear cell renal cell carcinoma
131 I- MIBG: 131 I- metaiodobenzylguanidine
OCT: Optical coherence tomography
